# Supplementary material for: Doubtful Clinical Value of Subtyping Anti-U1-RNP Antibodies Regarding the RNP-70 kDa Antigen in Sera of Patients with Systemic Lupus Erythematosus
Source: Int J Mol Sci. 2023 Jun 20;24(12):10398. doi: 10.3390/ijms241210398 (PMC10299012; doi:10.3390/ijms241210398)

Supplementary Figure S1: A box and whiskers plot (range, 25th to 75th percentile and median depicted) illustrating anti-U1-RNP and anti-RNP70 antibody levels in patients with SLE and MCTD testing positive for the antibodies. We observed a non-significant trend of higher anti-RNP70 antibody levels among subjects with MCTD compared to SLE.

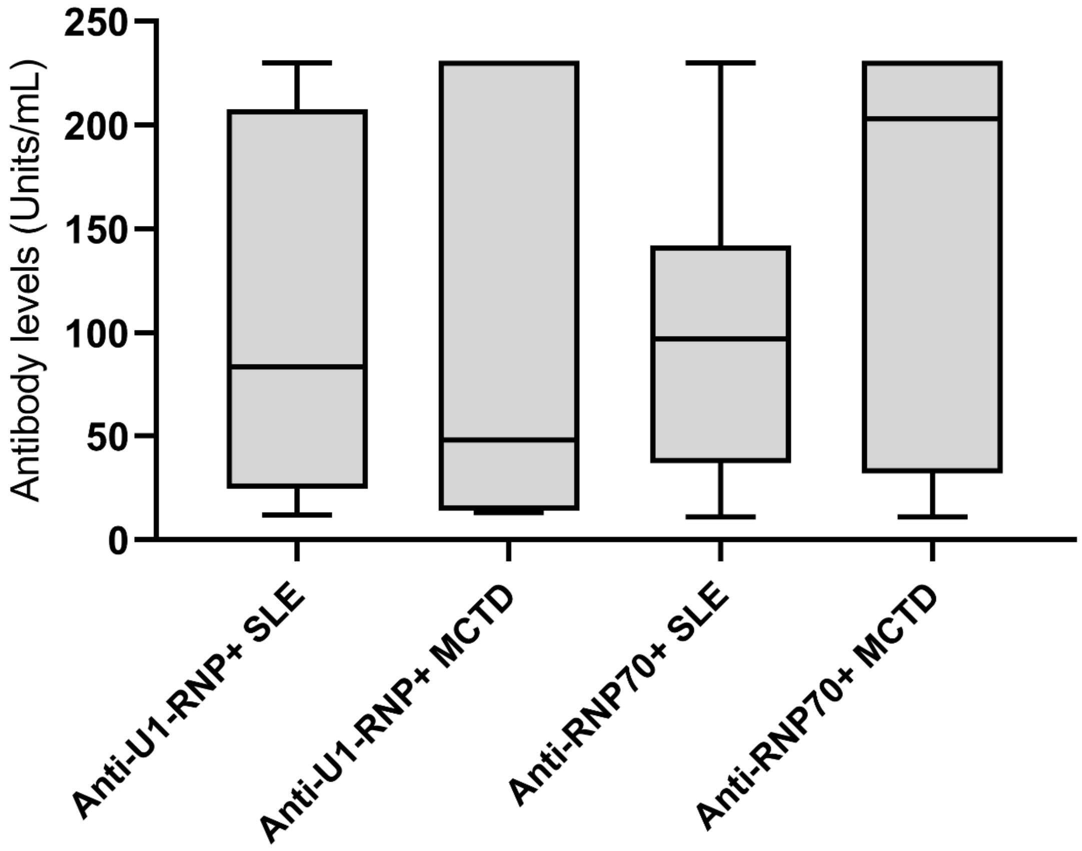

Supplement: Supplementary file 1 [file ijms-24-10398-s001.zip › ijms-2434761-supplementary.pdf]
